# Lumbar and lumbosacral spondylosis deformans in a crab-eating fox (*Cerdocyon thous*): a longitudinal radiographic case

**DOI:** 10.1007/s11259-026-11137-z

**Published:** 2026-03-19

**Authors:** Fabiane de Holleben Camozzato Fadrique, Eduarda Aléxia Nunes Louzada Dias Cavalcanti, Lorena Eduarda Feitosa Ferrarezi da Silva, Roberto Gumieiro Junior, Guilherme Albuquerque de Oliveira Cavalcanti, Raqueli Teresinha França

**Affiliations:** 1 Núcleo de Reabilitação da Fauna Silvestre, Veterinary College, Federal University of Pelotas, Capão do Leão, Rio Grande do Sul Brazil; 2 Department of Veterinary Clinics, Veterinary College, Federal University of Pelotas, Capão do Leão, Rio Grande do Sul Brazil

**Keywords:** Osteoarticular disorders, Wild canids, Diagnostic imaging, Degenerative vertebral disease, Lumbosacral pain, Serial radiography

## Abstract

Spondylosis deformans is a chronic degenerative disorder of the vertebral column that has been extensively described in domestic dogs but remains poorly documented in wild canids. The crab-eating fox (*Cerdocyon thous*) is frequently admitted to wildlife rehabilitation centers, and the characterization of musculoskeletal disorders in this species is particularly relevant given its ecological plasticity and close association with anthropized environments. This report describes a case of spondylosis deformans affecting the lumbar and lumbosacral spine of an adult neutered male *Cerdocyon thous* maintained under human care, which presented self-mutilation behavior and clinical signs consistent with lumbosacral pain. Three radiographic examinations of the vertebral column were performed in October 2021, October 2024 and January 2026, allowing longitudinal assessment of the lesion progression and stabilization patterns. The initial examination revealed ventral osteophyte formation, narrowing of intervertebral disc spaces, and vertebral endplate sclerosis involving the caudal lumbar vertebrae and the lumbosacral segment. At the follow-up examination, progression of the degenerative changes was evident, characterized by extensive ventral bony proliferation, greater involvement of the lumbosacral region, and further reduction of intervertebral disc spaces. Longitudinal comparison demonstrated a marked increase in both the extent and severity of the lesions over a five-year follow-up period, consistent with the chronic progression of spondylosis deformans. Serial radiography proved to be a valuable tool for the recognition and monitoring of degenerative vertebral disorders and may support clinical management of wild canids under human care.

## Background

Wild canids exhibit broad ecological adaptability and occupy both natural and anthropized environments, including agricultural and peri-urban areas. This close proximity to human activities increases their exposure to road traffic accidents, conflicts with domestic animals, and anthropogenic persecution, resulting in a growing demand for clinical care and diagnostic investigation, particularly in wildlife rehabilitation centers (Hanson et al. 2021; Schulz et al. 2024; Marques et al. 2024). In this context, the crab-eating fox (*Cerdocyon thous*) stands out as one of the most frequently admitted South American canids, owing to its wide geographic distribution and high tolerance of environmental modification (Cubas et al. 2014). Anatomical and radiographic studies have demonstrated a high degree of similarity between the axial skeleton of this species and that of the domestic dog, allowing, albeit with appropriate caution, the application of well-established clinical and radiographic concepts from small animal veterinary medicine to the assessment of vertebral disorders in *Cerdocyon thous* (Barisson et al. 2012).

Conventional radiography represents an essential diagnostic tool in wildlife clinical practice and is routinely employed in the investigation of musculoskeletal and neurological disorders, as well as in the follow-up of chronic conditions (Ribeiro et al. 2022). In *Cerdocyon thous*, retrospective studies conducted in rehabilitation centers have demonstrated a high prevalence of orthopedic conditions, particularly fractures associated with vehicular collisions, reinforcing the importance of systematic radiographic evaluation of the musculoskeletal system in this species (Pastor et al. 2021). However, structured descriptions of degenerative vertebral disorders in Neotropical wild canids remain scarce.

Spondylosis deformans is a chronic, non-inflammatory degenerative disorder characterized by marginal bony proliferation and, in advanced stages, the formation of bony bridges between adjacent vertebral bodies. It is frequently associated with segmental instability and intervertebral disc degeneration (Morgan et al. 1967; Germonpré et al. 2016; Seiler and Thrall 2024). From a morphological perspective, these proliferations correspond to enthesophytes related to the insertions of the annulus fibrosus and the longitudinal ligaments (Thomas and Fingeroth 2015). Although spondylosis deformans has been extensively described in domestic dogs, its occurrence in wild canids remains poorly documented, with isolated reports in *Vulpes vulpes* and *Canis latrans* (Harris 1977; Duckler 1997). Radiographic studies in dogs indicate a predilection for the lumbar and lumbosacral segments (Hadžijunuzović-Alagić et al. 2025), but comparable data for wild canids are limited.

In this context, the present report describes a case of spondylosis deformans affecting the lumbar and lumbosacral spine of an adult captive crab-eating fox (*Cerdocyon thous*), highlighting the associated clinical and radiographic findings and documenting the longitudinal radiographic progression of the lesions over a five-year period.

## Case presentation

### Initial presentation and first clinical evaluation (October 2021)

An adult male *Cerdocyon thous* was admitted to the Wildlife Rehabilitation Center of the Federal University of Pelotas (NURFS/UFPel), southern Brazil in 2018. In 2021, the animal underwent clinical evaluation due to intense self-mutilation behavior characterized by licking, biting, and cutaneous excoriations predominantly affecting the lumbosacral region, associated with marked pruritus. On general physical examination at initial presentation, the animal was alert and responsive, with pink mucous membranes and normal hydration status. Heart rate, respiratory rate and rectal temperature were within the expected physiological ranges for the species. Based on the pattern and distribution of the skin lesions, the case was initially managed as a dermatological condition, with a clinical suspicion of flea allergy dermatitis. Areas of alopecia, erythema, crusting, and self-inflicted traumatic lesions were observed on the dorsal surface, particularly over the lumbosacral region. Despite dermatological management, including ectoparasite control through topical application of fipronil (spot-on), combined with local cleansing and topical wound-healing therapy, only partial improvement of the cutaneous lesions and reduction in pruritus were observed, while the self-mutilation behavior persisted.

Concurrently, conservative supportive therapy was instituted for pain and pruritus control, with administration of dipyrone (25 mg/kg, intramuscularly, twice a day, for seven days), tramadol hydrochloride (10 mg/kg, orally, once a day, for five days), and dexamethasone (0.14 mg/kg, intramuscularly, once a day, for nine days), proving effective against the acute crisis of clinical signs.

Given the incomplete response to dermatological and analgesic treatments and the persistence of self-mutilation behavior localized to the lumbosacral region, the diagnostic investigation was expanded to include neurological assessment and imaging studies of the vertebral column. On physical and neurological examination, increased pain sensitivity was evident on palpation of the lumbosacral region, associated with proprioceptive deficits in the pelvic limbs. No concurrent systemic abnormalities were detected on physical and neurological examination, and subsequent laboratory investigations did not reveal evidence of systemic, infectious, or metabolic disease. The animal was in a stable general condition at the time of evaluation. Self-mutilation behavior directed toward the lumbosacral region was active at the time of examination and had persisted despite initial clinical management.

Based on these findings, the patient was referred for radiographic evaluation of the vertebral column at the Diagnostic Imaging and Cardiology Laboratory of the Federal University of Pelotas (LADIC/UFPel). The examinations were performed under appropriate sedation and restraint, following an anesthetic protocol consisting of intramuscular morphine (0.4 mg/kg) and intravenous ketamine (2 mg/kg) combined with midazolam (0.2 mg/kg), complemented by a bolus of propofol (2 mg/kg). No complications were observed during the procedure.

Three radiographic examinations of the lumbar and lumbosacral spine were performed, with intervals of approximately three and two years between them: the first in October 2021, the second in October 2024, and the third in January 2026. Radiographs were obtained using a standardized digital radiography system with fine local spot, with exposure parameters of approximately 81 kVp and 10 mAs, adjusted according to body size and anatomical region. Standardized positioning in ventrodorsal and right and left laterolateral projections was used, aiming to minimize anatomical superimposition and ensure reproducibility of the radiographic findings, allowing standardized comparison between examinations, following established technical principles and evaluation criteria described for radiographic examination of the axial skeleton in small animals (Mühlbauer and Kneller 2024). The severity of spondylosis deformans was graded according to the four-stage radiographic classification of vertebral osteophytes described by Morgan et al. (1967), in which first-degree lesions correspond to small marginal bony spurs, second-degree lesions to larger “parrot’s beak” osteophytes, third-degree lesions to osteophytes extending beyond the vertebral endplates without fusion, and fourth-degree lesions to complete ventral bony fusion between adjacent vertebral bodies. In the present case, first- and second-degree lesions were considered mild to moderate, third-degree lesions moderate to severe, and fourth-degree lesions severe ankylosing spondylosis deformans.

In order to mitigate post-handling discomfort during the radiographic evaluation, carprofen was administered (4.4 mg/kg, orally, once a day, for seven days), followed by the prescription of gabapentin (15 mg/kg, orally, once a day, for 30 days) for chronic pain control, which was effective in maintaining the patient’s comfort.

### Clinical investigation and first radiographic evaluation (October 2021)

At the time of initial clinical evaluation, the animal had a body weight of 8.7 kg and a body condition score of 5 (1–5), which were recorded because excessive body weight may represent a relevant biomechanical factor contributing to spinal overload and progression of degenerative vertebral disease.

**Fig. 1:**
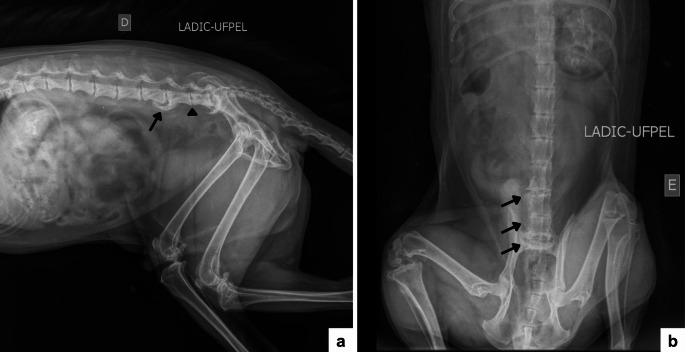
Radiographic images of the lumbar and lumbosacral spine of an adult male *Cerdocyon thous*, obtained in October 2021. **a** Right laterolateral projection showing the presence of deforming spondylosis between L5–L6 and L7–S1 (black arrow), as well as decreased intervertebral space between L6–L7 with mild sclerosis of the endplates (black arrowhead). **b** Ventrodorsal projection showing lateral osteophytes between L5–L6, L6–L7, and L7–S1 (black arrows)

On radiographic evaluation, ventral osteophytes were observed involving the vertebral bodies of L5–L6 and L6–S1, associated with narrowing of the intervertebral disc spaces between L5–L6, L6–L7, and L7–S1. Vertebral endplate sclerosis was also identified adjacent to the intervertebral disc spaces L4–L5, L5–L6, and L6–L7, as well as sclerosis of the caudal surface of the L7 vertebral body associated with a proliferative change on the cranial surface of the first sacral vertebra (Fig. 1). No relevant differences were observed between the right and left laterolateral projections, and the radiographic findings were consistent across both views. Overall, the alterations were compatible with mild to moderate spondylosis deformans, characterized by ventral marginal bony proliferations without complete bony bridging between adjacent vertebral bodies.

### Clinical follow-up and second radiographic evaluation (October 2024)

At clinical follow-up, the body weight was 6.5 kg and the body condition score was 4 (1–5), which were recorded to allow longitudinal assessment of the possible influence of body weight variation on the progression of the vertebral degenerative process.

**Fig. 2:**
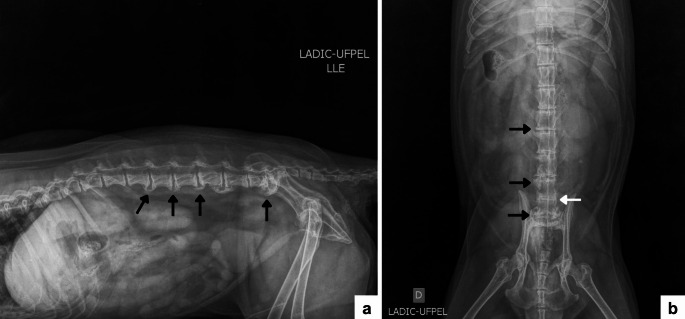
Radiographic images of the lumbar and lumbosacral spine of an adult male *Cerdocyon thous*, obtained in October 2024. **a** Right laterolateral projection showing progression of deforming spondylosis between L5–L6 and L7–S1, as well as development of new spondyloses between L2–L3, L3–L4, and L4–L5. **b** Ventrodorsal projection showing exuberant osteophytes between L3–L4, L5–L6, and L7–S1 (black arrows), with marked reduction of the intervertebral space between L6–L7 (white arrow)

Progression of the previously described alterations was evident, characterized by extensive ventral and caudoventral bony proliferations involving the vertebral bodies from L2 to S1. The changes were more pronounced in the lumbosacral segment, particularly between L7 and S1, where exuberant bony proliferation was associated with vertebral endplate sclerosis and marked narrowing of the intervertebral disc spaces, especially between L5 and S1 (Fig. 2). The distribution, severity, and morphological characteristics of the radiographic findings observed in 2024 indicated chronic progression of the degenerative process affecting the vertebral column. Compared with the previous examination, the condition had progressed to moderate to severe spondylosis deformans, with extensive marginal bony proliferations involving multiple intervertebral spaces and a tendency toward the formation of ventral bony bridges, particularly in the lumbosacral segment. Longitudinal comparison between the two examinations demonstrated a marked increase in both the extent and severity of the structural alterations over a five-year period, temporally associated with the persistence of neurological signs.

### Clinical follow-up and third radiographic evaluation (January 2026)

At clinical follow-up, the animal was classified as geriatric and had a body weight of 10.35 kg and a body condition score of 5 (1–5), a marked increase in body weight that may have contributed to mechanical overload of the ankylosed vertebral segments, although no overt clinical signs of pain were observed at that time.

**Fig. 3:**
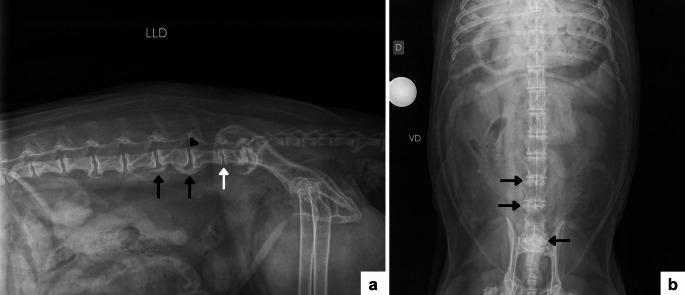
Radiographic images of the lumbar and lumbosacral spine of an elderly male *Cerdocyon thous*, obtained in January 2026. **a** Right laterolateral projection showing advanced deforming spondylosis (black arrows) between vertebrae L2–L3, L3–L4, L4–L5, L5–L6, and L7–S1. Mild narrowing of the L6–L7 intervertebral space (white arrow) and subtle misalignment between L5–L6 vertebrae (black arrowhead) are also observed. **b** Ventrodorsal projection showing lateral osteophytes (black arrows)

Radiography of the thoracolumbar, lumbar, and lumbosacral spine (right lateral and ventrodorsal projections) revealed ankylosing spondylosis between L2–L3, L3–L4, L4–L5, L5–L6, and L7–S1, associated with intervertebral disc space narrowing at L6–L7 (mild) and L7–S1 (more evident), and vertebral endplate sclerosis affecting the caudal aspect of L7 and the cranial aspects of L6 and S1. A subtle vertebral step/misalignment at L5–L6 was also noted. Overall, these findings were consistent with further progression of the chronic degenerative process affecting the lumbar and lumbosacral spine (Fig. 3).

At the time of the last follow-up evaluation, the animal was not receiving any continuous analgesic or anti-inflammatory treatment and did not exhibit overt clinical signs of pain, discomfort, or self-mutilation behavior. Palpation of the lumbosacral region elicited no marked pain response, and the patient remained clinically stable under routine management conditions.

### Complementary laboratory investigation and clinical outcome (October 2021–January 2026)

Additional laboratory tests were performed, such as complete blood count (Table 1), reticulocyte count (Table 1), serum biochemistry (Table 2) and screening for hemoparasites by peripheral blood smear at all time points, all of which were negative. Coproparasitological examination and specific dermatological tests were not performed.

**Table 1:** Serial hematological parameters of an adult male Cerdocyon thous during long-term follow-up, with lumbar and lumbosacral spondylosis deformans

Parameter	Oct 2021	Sep 2024	Jun 2025	Jan 2026	Reference range*
RBC (×10⁶/µL)	5,15	4,86	3,87	3,24	3,05–6,08
Hemoglobin (g/dL)	14,4	15	11,9	10,3	10–18,1
Hematocrit (%)	44.0	42,7	33,9	28,5	28–53
MCV (fL)	85,4	87,9	87,6	87,8	79,11–100
MCHC (%)	32,7	35,1	35,2	36,2	30,22–38,46
Platelets (×10³/µL)	313	281	274	217	18–636
Metarrubricytes (%)	0	0	5	0	0–1
WBC (/µL)	11.000	13.300	7.100	2.100	3.400–23.200
Neutrophils (/µL)	6.600	7.315	3.834	1.134	1.146–12.990
Band neutrophils (/µL)	0	0	0	0	0–700
Lymphocytes (/µL)	2.530	3.325	1.136	714	210–3.990
Monocytes (/µL)	110	665	142	42	40–2.550
Eosinophils (/µL)	1.760	1.995	1.988	210	270–3.940
Basophils (/µL)	0	0	0	0	0–520
Total protein (g/dL)	7	8	6	6,8	4,6–9,4
Fibrinogen (mg/dL)	200	400	200	200	100–600
Reticulocytes (%)	-	-	1,8	4	-
Reticulocytes (/µL)	-	-	69.660	129.600	-

* Reference ranges from Mattoso et al. (2012)

**Table 2:** Serial serum biochemical parameters of an adult male Cerdocyon thous during long-term follow-up, with lumbar and lumbosacral spondylosis deformans

Parameter	Oct 2021	Sep 2024	Jun 2025	Jan 2026	Reference range*
Albumin (g/dL)	3,16	2,87	-	-	2–6,2
ALP (UI/L)	26,6	9,8	16,5	23	8,2–267
ALT (UI/L)	29,8	20,2	24,5	37,8	2,6–231,5
AST (UI/L)	36	-	17,1	26	-
Cholesterol (mg/dL)	159,67	188,33	135,58	115	-
CK (UI/L)	801,2	-	127	485,5	-
Creatinine (mg/dL)	0,7	0,7	0,7	0,93	0,5–1,5
GGT (UI/L)	-	N/D	2,6	-	0,6–5,7
Glucose (mg/dL)	77,87	115,39	96,4	-	-
LDH (UI/L)	-	-	-	313	-
Triglycerides (mg/dL)	103,26	46,66	47,8	132	-
Urea (mg/dL)	62,02	32,4	32,27	32	22–87

* Reference ranges from Mattoso et al. (2012). N/D: Not detectable, concentration below the test’s detection limit (3 IU/L)

During clinical follow-up, the self-mutilation behavior showed progressive reduction following the implementation of conservative supportive therapy, without the need for continuous use of an Elizabethan collar and with adequate healing of the cutaneous lesions previously observed in the lumbar region. Based on the long-term follow-up, the structural prognosis was considered guarded due to the progressive degenerative nature of the disease, whereas the functional prognosis regarding pain control and self-mutilation behavior was considered moderately favorable under conservative management.

All procedures corresponded exclusively to routine clinical care and diagnostic investigation, conducted in accordance with institutional animal welfare guidelines. Ethical approval by an institutional animal use ethics committee was not required for this type of clinical case.

## Discussion and conclusions

Although spondylosis deformans has been extensively described in companion animal veterinary medicine, it remains poorly documented in wild canids, particularly in Neotropical species, with available information largely restricted to isolated case reports (Harris 1977; Duckler 1997; Germonpré et al. 2016). In this context, the present report is noteworthy for documenting an axial degenerative disorder in *Cerdocyon thous* with longitudinal radiographic follow-up over a five-year period, allowing correlation between structural progression, clinical signs, and therapeutic response in a wild canid maintained under human care.

In wildlife rehabilitation centers, wild canids are frequently subjected to clinical and imaging evaluations due to their high exposure to anthropogenic injuries, particularly vehicular collisions and polytrauma (Schulz et al. 2024; Marques et al. 2024). *Cerdocyon thous* is among the species most commonly admitted and subjected to radiographic evaluation in these settings (Cubas et al. 2014; Ribeiro et al. 2022). Nevertheless, while fractures and traumatic injuries represent the most frequently reported conditions in this species (Pastor et al. 2021; Marques et al. 2024), chronic degenerative disorders of the axial skeleton tend to be underreported, either due to lower clinical suspicion or to the scarcity of longitudinal follow-up.

Available data on spondylosis deformans in wild canids are largely limited to isolated case descriptions, such as those reported in *Vulpes vulpes* (Harris 1977) and a case in *Canis latrans* associated with significant vertebral trauma (Duckler 1997), as well as descriptions from archaeological and paleopathological contexts demonstrating the occurrence of osteodegenerative changes across different ecological and historical settings (Rothschild et al. 2001; Germonpré et al. 2016). To the best of our knowledge, this represents the first documented case of spondylosis deformans in *Cerdocyon thous*, with the additional distinction of serial radiographic follow-up, a feature rarely available in studies of wild canids, particularly in captive individuals.

In the present case, the initial radiographic evaluation revealed ventral marginal bony proliferations affecting the caudal lumbar and lumbosacral segments, associated with narrowing of multiple intervertebral disc spaces and vertebral endplate sclerosis. These findings are consistent with classical descriptions of spondylosis deformans in dogs, in which early changes include marginal enthesophytes or osteophytes frequently associated with segmental instability and intervertebral disc degeneration (Morgan et al. 1967; Thomas and Fingeroth 2015; Seiler and Thrall 2024). The predominant distribution between L5 and S1 is also consistent with the small animal literature, which recognizes the lumbosacral junction as a region of increased biomechanical stress and, consequently, greater susceptibility to degenerative changes (De Risio et al. 2000; Ness 1994; Hadžijunuzović-Alagić et al. 2025). In *C. thous*, anatomical interpretation of this region must also consider specific features of the axial skeleton, such as the presence of seven lumbar vertebrae and a sacrum formed by two fused vertebrae, as described by Barisson et al. (2012), which is relevant for standardizing vertebral counting and topographic description in serial radiographic examinations.

From a pathophysiological perspective, intervertebral disc degeneration involves biochemical and structural alterations that reduce load-absorbing capacity and lead to abnormal force redistribution along the vertebral column, favoring micromotion and chronic stress at the insertions of the annulus fibrosus and the longitudinal ligaments (Coates 2000). This process triggers a marginal osteoproliferative response through endochondral ossification, typically exhibiting ventral and lateral growth, a pattern characteristic of spondylosis deformans (Thomas and Fingeroth 2015; Seiler and Thrall 2024). In the present case, the radiographically observed narrowing of intervertebral disc spaces and vertebral endplate sclerosis support the hypothesis of chronic disc degeneration as a central component of the disease process, with structural progression clearly documented over a five-year period.

The radiographic progression observed at the most recent examination (2024), with extension of bony proliferations to more cranial segments (L2 to S1) and greater involvement of the lumbosacral region, reinforces the chronic, cumulative, and progressive nature of spondylosis deformans, as described in dogs, in which the disease typically evolves slowly, particularly in older individuals (Coates 2000; Thomas and Fingeroth 2015; Seiler and Thrall 2024). In wild animals maintained under human care, increased life expectancy and the possibility of continued monitoring enhance the likelihood of detecting degenerative changes that might remain subclinical or undocumented under free-ranging conditions (Cubas et al. 2014; Germonpré et al. 2016; Latham and Losey 2019). Comparative studies further indicate that spondylosis deformans occurs in both domestic dogs and wild canids, with multifactorial influences and greater frequency and/or severity in captive individuals, possibly related to longevity and environmental conditions (Latham and Losey 2019).

At the final radiographic examination performed in January 2026, the presence of extensive ventral bony bridges and ankylosis involving multiple lumbar and lumbosacral segments represented the advanced stage of the degenerative process. In dogs, the formation of complete ventral bony bridges corresponds to late-stage spondylosis deformans and reflects long-standing disc degeneration and chronic segmental instability (Morgan et al. 1967; Thomas and Fingeroth 2015; Seiler and Thrall 2024). In the present case, the progression from marginal osteophytes to multilevel ankylosis over a five-year period provides rare longitudinal documentation of the natural history of spondylosis deformans in a wild canid, reinforcing the chronic and cumulative nature of this disorder under captive conditions.

Notably, despite the marked radiographic progression and extensive multilevel ankylosis documented in January 2026, the animal did not exhibit overt pain-related behaviors at the last follow-up and was not receiving continuous analgesic or anti-inflammatory treatment. This apparent dissociation between advanced structural changes and current clinical expression is compatible with the biomechanical consequences of late-stage spondylosis deformans. In advanced disease, marginal osteoproliferation may progress to ventral bony bridging and partial or complete fusion between adjacent vertebral bodies, which reduces segmental flexibility and abnormal micromotion, thereby decreasing chronic stress at ligamentous and annulus fibrosus insertions and potentially reducing nociceptive stimulation, functioning analogously to a spontaneous vertebral arthrodesis (Thomas and Fingeroth 2015; Seiler and Thrall 2024). Nevertheless, severe spondylosis may coexist with other degenerative processes (e.g., disc degeneration, facet disease or degenerative lumbosacral stenosis) and conventional radiography cannot directly assess neural compression; therefore, clinical interpretation may remain integrated with the neurological examination and longitudinal follow-up (Meij and Bergknut 2010; Worth et al. 2019; Seiler and Thrall 2024).

Serial hematological, serum biochemical and parasitological evaluations did not reveal evidence of a primary infectious, inflammatory or systemic disease underlying the clinical presentation. Most hematological and biochemical parameters remained within or close to the reference intervals established for captive *Cerdocyon thous*. Mild variations observed over time, such as progressive decreases in erythrocyte parameters and fluctuations in leukocyte counts, were interpreted as compatible with aging, chronic disease and physiological variability rather than with an active inflammatory or infectious process, in the absence of compatible clinical signs. Importantly, peripheral blood smears performed at all time points were consistently negative for hemoparasites, reducing the likelihood of vector-borne infections as a cause of pruritus, behavioral changes or systemic deterioration. Together, these findings support the interpretation that the clinical signs were primarily associated with a chronic degenerative vertebral disorder rather than with an underlying hematological, metabolic or infectious disease (Mattoso et al. 2012).

Age represents a central factor in interpreting the progression of the observed changes. The animal was admitted to the rehabilitation center in 2018 already as an adult; thus, at the time of the first radiographic examination in 2021, it was considered a mature adult. At the second examination in 2024, the elapsed time since admission, together with the life expectancy described for *Cerdocyon thous* in captivity, allowed classification of the individual as geriatric (Cubas et al. 2014). In domestic canids, spondylosis deformans shows a consistent association with aging, reflecting a cumulative degenerative process with slow progression, most frequently identified in long-lived individuals (Coates 2000; Thomas and Fingeroth 2015; Seiler and Thrall 2024). Accordingly, the longevity achieved by this animal under human care may have been a determining factor for the clinical and radiographic manifestation of degenerative changes that might not have been detected under free-ranging conditions (Germonpré et al. 2016; Latham and Losey 2019).

In addition, body condition and weight variation observed during follow-up may have influenced disease evolution. At the 2021 examination, the animal weighed 8.7 kg, a value at the upper limit of the range described for the species (Cubas et al. 2014). A reduction to 6.5 kg was observed in 2024, followed by a marked increase to 10.35 kg in 2026. In dogs, increased biomechanical load on the lumbosacral segment associated with higher body weight is recognized as a predisposing factor for segmental instability and disc degeneration (Ness 1994; De Risio et al. 2000; Levine et al. 2006; Worth et al. 2019). Although a direct causal relationship cannot be established, the periods of obesity observed at the initial and final evaluations may have contributed to increased mechanical overload of the lumbosacral region.

From a clinical standpoint, pain elicited on lumbosacral palpation and proprioceptive deficits in the pelvic limbs observed in this patient are consistent with degenerative involvement of the L7–S1 segment and possible compromise of nerve roots (cauda equina). In dogs, lumbosacral pain is often the primary clinical sign in degenerative disorders affecting this region and may occur even in the absence of marked neurological deficits (Ness 1994; De Risio et al. 2000; Meij and Bergknut 2010; Worth et al. 2019). Lesions or compressive processes at the lumbosacral junction may result in pain, postural alterations, and proprioceptive deficits due to nerve root involvement, with variable severity depending on the degree and nature of the compromise (De Lahunta et al. 2021). Thus, although spondylosis deformans may represent an incidental finding, its clinical relevance increases when associated with disc degeneration, degenerative lumbosacral stenosis, facet joint disease, or foraminal narrowing, which may culminate in chronic pain and neurological signs (De Risio et al. 2000; Levine et al. 2006; Thomas and Fingeroth 2015; Worth et al. 2019).

The self-mutilation behavior initially directed toward the lumbar region is plausible in the context of chronic and/or neuropathic pain. In small animals, irritation of nerve roots, intermittent compression, and central sensitization may manifest as persistent licking, biting, and self-trauma behaviors (De Risio et al. 2000; Ness 1994; Severo et al. 2007). The temporal correlation between clinical signs, radiographic progression, and response to conservative management supports the conclusion that the axial degenerative process was clinically relevant in this patient, even though conventional radiography does not allow direct inference of the degree of neural compression.

Regarding differential diagnoses, dermatological conditions became less likely due to the absence of primary skin lesions and evidence of active pruritus or ectoparasite infestation, while extra-vertebral orthopedic causes were ruled out by the absence of pain on coxofemoral or sacroiliac manipulation. Furthermore, characteristic patterns of inflammatory spondyloarthropathies differ from the degenerative proliferative pattern observed in this case, and paleopathological discussions of these conditions help reinforce such morphological distinctions in the differential diagnosis of axial skeletal disorders (Rothschild et al. 2001).

Therapeutic management of spondylosis deformans and degenerative lumbosacral disorders is generally conservative and multimodal, focusing on analgesia, inflammatory modulation, weight control, and rehabilitation strategies, with surgical intervention reserved for selected cases (Meij and Bergknut 2010; Worth et al. 2019; Seiler and Thrall 2024). In the present case, the progressive reduction in self-mutilation behavior and clinical stabilization suggest a beneficial response to conservative management. Structured rehabilitation approaches have been described as relevant components of multimodal treatment in small animals (Memon and Xie 2023) and are particularly important in captive wild animals, in which invasive interventions may be limited by welfare considerations (Cubas et al. 2014).

From a diagnostic perspective, conventional radiography proved fundamental for identifying and monitoring structural progression over a five-year period. Although it has limitations in the direct assessment of neural compression and soft tissues, radiography remains widely used as a screening and follow-up tool, requiring interpretation integrated with clinical and neurological examinations (Thomas and Fingeroth 2015; Worth et al. 2019; Seiler and Thrall 2024). Technical standardization and proper positioning are essential for reproducibility in serial examinations (Mühlbauer and Kneller 2024; Seiler and Thrall 2024), and retrospective studies in dogs reinforce that degenerative vertebral disorders are among the most frequent radiographic findings in clinical practice (Santos et al. 2006). In addition, emerging diagnostic support tools, such as deep learning models applied to radiographic imaging, highlight the ongoing and evolving role of radiography in the detection and monitoring of spondylosis deformans (Park et al. 2024).

The correlation between clinical signs, radiographic findings, and therapeutic response supported a presumptive diagnosis of spondylosis deformans with lumbosacral involvement in this crab-eating fox (Cerdocyon thous), with rare longitudinal documentation of lesion progression over a five-year period. This case highlights the relevance of longitudinal radiographic monitoring for the recognition and follow-up of chronic degenerative vertebral disorders in wild canids under human care. Beyond the traditional focus on traumatic injuries, axial degenerative conditions should be considered important differential diagnoses in captive wild canids presenting chronic pain-related behaviors, contributing to improved clinical management, welfare, and quality of life.

In conclusion, this report provides a rare longitudinal radiographic documentation of spondylosis deformans in Cerdocyon thous, with serial follow-up over a five-year period and correlation with clinical signs and therapeutic response. The case highlights the relevance of axial degenerative disorders as a cause of chronic pain-related behaviors in captive wild canids and underscores the value of standardized radiographic monitoring in long-term management. Important limitations include the absence of advanced imaging, such as magnetic resonance imaging, precluding direct assessment of neural compression and degenerative lumbosacral stenosis, and the inherent constraints of a single-case report. Nevertheless, this case contributes original data to the scarce literature on degenerative vertebral disease in Neotropical canids and may serve as a reference for future clinical and pathological investigations in this group

## Data Availability

No datasets were generated or analyzed during the current study.
